# Incarcerated Appendix Epiploica in Inguinal Hernia Sac: Treatment with Laparoscopic TAPP Approach—Report of a Rare Case

**DOI:** 10.1155/2020/4178523

**Published:** 2020-02-21

**Authors:** Abdullah Yildiz

**Affiliations:** Department of General Surgery, Umraniye Education and Research Hospital, Istanbul, Turkey

## Abstract

Appendix epiploica (AE) in an incarcerated inguinal hernia sac is very rare. We herein report the case of a 57-year-old man admitted to the emergency department with complaints of nausea, swelling, and pain in the left inguinal area. He was diagnosed with left incarcerated inguinal hernia and treated laparoscopically with transabdominal preperitoneal (TAPP) mesh hernioplasty. During the operation, AE, lodged in the direct hernia sac, was seen to originate from the sigmoid colon. The narrow internal inguinal ring was incised at the 2 o'clock position using a monopolar hook, and the hypertrophic AE was reduced to the abdomen and resected. The patient was discharged uneventfully on the second postoperative day.

## 1. Introduction

Irreducible or incarcerated omentum and intestinal loops are frequently found in the inguinal hernia sac. Rarely, AE, adnexa, and appendix vermiformis can also be detected in the incarcerated hernia sac. AE in the inguinal hernia sac is rare, with very few cases reported in the literature [1].

In this case report, we investigated the role of laparoscopic TAPP for the emergency treatment of inguinal hernia with AE incarceration, the latter of which was diagnosed during the TAPP procedure itself.

## 2. Case Report

A 57-year-old man was admitted to the emergency department with complaints of swelling and pain in the left inguinal area. He had nausea but no vomiting. On physical examination, a painful, irreducible mass of about 2 cm to 3 cm in size was palpated in the left inguinal area; no other abnormality was detected. Laboratory tests revealed leukocytosis. Other laboratory and biochemical tests were normal. Edematous, hypertrophic adipose tissue was identified in the hernia sac during abdominal ultrasonography as ultrasonographic confirmation was adequate for diagnosis. We planned a laparoscopic TAPP surgery considering laparoscopy's superiority to the open technique for visualization of the hernia contents.

**Figure 1:**
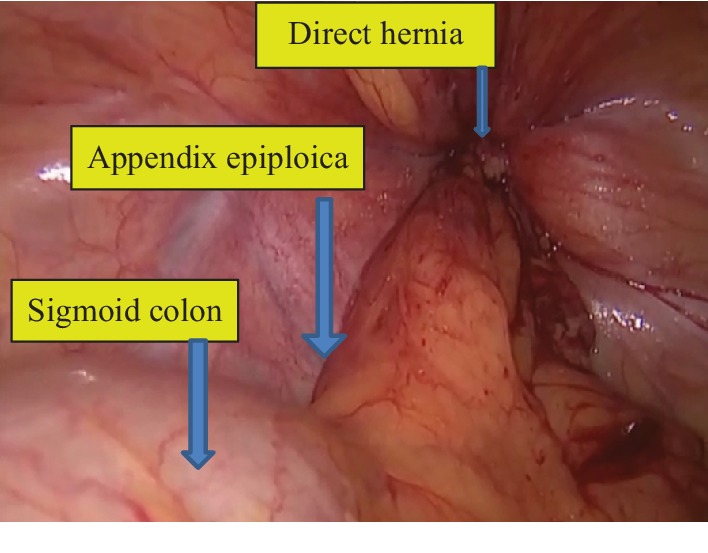
Appendix epiploica in the direct hernia sac.

**Figure 2:**
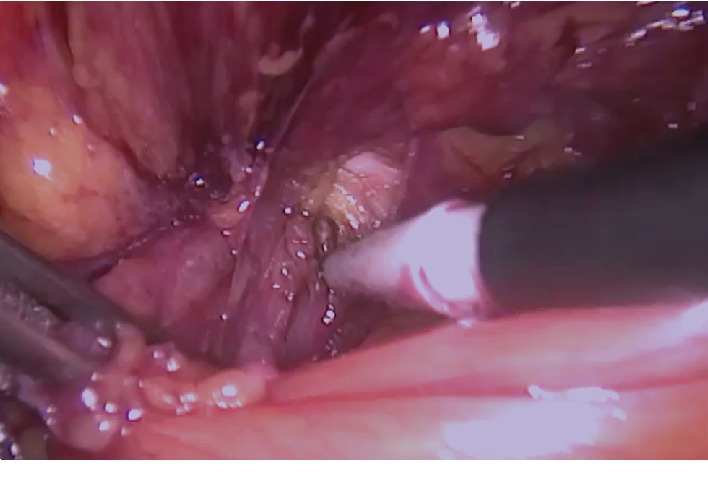
Internal ring incised and enlarged with hook.

**Figure 3:**
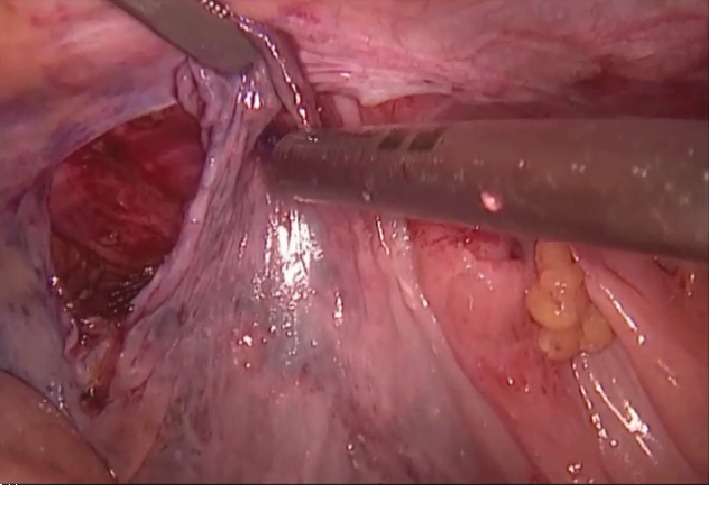
Peritoneal closure with tacker.

With the patient under general anesthesia, a 12 mm trocar was inserted directly under the umbilicus, and the abdomen was insufflated. Two 5 mm trocars were inserted through the right and left midclavicular lines at the level of the umbilicus. During exploration, AE of the sigmoid colon was observed in the left hernia sac (Figure 1). Other abdominal organs were normal. The contents could not be reduced to the abdomen despite external compression and internal traction. The narrow inner ring, about 10 mm to 15 mm in diameter, was incised and enlarged at the 2 o'clock position by hook cautery (Figure 2). Preperitoneal fatty tissue was excised along with the hypertrophic AE. The sigmoid colon appeared normal. The direct hernia sac was retracted using a grasper and fixed to Cooper's ligament with a tacker. A 15 cm × 12 cm polypropylene mesh was fixed with the absorbable tacker, and the peritoneal opening was closed (Figure 3). The patient was uneventfully discharged from the hospital on the second postoperative day and remained symptom-free during follow-up visits. The histopathological examination did not reveal any malignancy.

## 3. Discussion

Epiploic appendices (EA) are small, adipose structures covered by the colon serosa. Measuring 1 cm to 2 cm in width and 2 cm to 5 cm in length, EA can be observed on the peritoneum, small intestine, and, most commonly, throughout the colon, except for the rectum [2]. The EA are distributed throughout the colon in two parallel lines between the tenia coli, being firmly adhered to the serous intestinal surface. They are more often found in the sigmoid (57%), cecum (26%), ascending (9%), transverse (6%), and descending colon (2%) [3]. Although their function is uncertain, EA have been reported to assist in colonic peristalsis and colonic absorption, in addition to having omentum-like immunity-related functions [1]. Within the hernia sac, AE may be hypertrophic, edematous, necrotic, or inflamed. AE-related conditions, such as torsion-induced necrosis, calcification after aseptic fat necrosis, and primary and secondary inflammation, may present with clinical features resembling acute appendicitis and diverticulitis [4]. Recently, few case reports on this topic have been published [4, 5]. But, only Jain et al. reported one such case on which laparoscopic TAPP was performed [6]. While many studies report sigmoid colon AE lodged within left inguinal hernia sac, there have been case reports where AE were seen in the right inguinal hernia sac [7]. Besides the commonly observed omentum or bowel loops, the hernia sac may also contain appendix vermiformis, Meckel's diverticulum, uterus, ovaries, fallopian tubes, AE, lipoma, or other organs [7]. It is well known that the most common content of a strangulated inguinal hernia is a small bowel loop or part of the major omentum. Certain parts of the colon, urinary bladder, vermiform appendix, Meckel's diverticulum, uterine tube and ovary, and other tissues as AE may be observed in a strangulated groin hernia much less frequency [1].

It is possible to come upon unexpected structures within the irreducible inguinal hernia sac. Therefore, a detailed anamnesis and physical examination, together with radiological and laboratory tests, will help the surgeon to plan the surgery. In this case, we planned a laparoscopic operation due to its diagnostic and treatment-related advantages over open laparotomy. Despite advanced imaging tools, contents of the hernia sac cannot always be accurately identified preoperatively. The contents of the hernia sac may get reduced to the abdomen during anesthetic induction or surgery, in which case laparotomy may be necessary to observe and check the contents of the sac. While laparotomy increases the risks of postoperative wound infection and incisional hernia, failing to notice ischemic or necrotic bowel contents by not performing laparotomy will have serious postoperative consequences [8]. Therefore, in cases of irreducible hernia, it may be necessary to explore the hernia sac before incising the external oblique fascia.

Our aim was to investigate the role of laparoscopic procedures in the emergency treatment of inguinal hernia. A longer learning curve, increased risk of iatrogenic organ injury during the reduction of hernia content, and technical difficulty compared with the open technique are limiting reasons for TAPP [8]. However, the use of laparoscopy in elective hernia surgery is increasing. Easier observation of abdominal hernia content, decreased risk of adhesions, early discharge, lower wound infection rate, and shorter recovery period are some of the advantages of TAPP. Therefore, we believe that laparoscopic surgery for emergency inguinal hernias is beneficial provided that a surgeon has enough experience.

## 4. Conclusion

Incarcerated inguinal hernia with AE content is rarely reported in the literature. To the best of our knowledge, this is the second case report of laparoscopic TAPP hernioplasty in an emergency setting. The report also brings the treatment of some rare diseases with laparoscopic TAPP to the attention of the academic community. In addition to elective hernia surgery, laparoscopic TAPP hernioplasty can also be safely performed in irreducible or incarcerated inguinal hernia, such as this case of incarcerated AE.
